# Leveraging a Multi-Omics Strategy for Prioritizing Personalized Candidate Mutation-Driver Genes: A Proof-of-Concept Study

**DOI:** 10.1038/srep17564

**Published:** 2015-12-03

**Authors:** Keyue Ding, Songfeng Wu, Wantao Ying, Qi Pan, Xiaoyuan Li, Dachun Zhao, Xianyu Li, Qing Zhao, Yunping Zhu, Hong Ren, Xiaohong Qian

**Affiliations:** 1 Institute for Viral Hepatitis, Key Laboratory of Molecular Biology for Infectious Diseases, Ministry of Education of China; Depeartment of Infectious Diseases, The Second Affiliated Hospital of Chongqing Medical University, Chongqing, P. R. China, 400010; 2 State Key Laboratory of Proteomics, National Protein Science Beijing Center, Beijing Proteome Research Center, Beijing Institute of Radiation Medicine, Beijing, P.R. China, 102206; 3 Department of Medical Oncology, Peking Union Medical College Hospital, Chinese Academy of Medical Sciences and Peking Union Medical College, Beijing, P. R. China, 100730; 4 Department of Pathology, Peking Union Medical College Hospital, Chinese Academy of Medical Sciences and Peking Union Medical College, Beijing, P. R. China, 100730

## Abstract

The expression of mutant forms of proteins (e.g., oncogenes and tumor suppressors) has implications in cancer biology and clinical practice. Initial efforts have been made to characterize the transcription of tumor-mutated alleles; however, few studies have been reported to link tumor-mutated alleles to proteomics. We aimed to characterize the transcriptional and translational patterns of tumor-mutated alleles. We performed whole-exome sequencing, RNA-seq, and proteome profiling in a hyper-mutated patient of hepatocellular carcinoma. Using the patient as a model, we show that only a small proportion of tumor-mutated alleles were expressed. In this case, 42% and 3.5% of the tumor-mutated alleles were identified to be transcribed and translated, respectively. Compared with genes with germline variations or without mutations, somatic mutations significantly reduced protein expression abundance. Using the transcriptional and translational patterns of tumor-mutated alleles, we classified the mutations into four types, and only one type may be associated with the liver cancer and lead to hepatocarcinogenesis in the patient. Our results demonstrate how tumor-mutated alleles are transcribed and translated, and how the expression enables the classification of somatic mutations that cause cancer. Leveraging multiple ‘omics’ datasets provides a new avenue for understanding patient-specific mutations that underlie carcinogenesis.

With the advent of next-generation sequencing, many cancer-genome sequencing studies have been conducted, e.g., the Cancer Genome Atlas (TCGA), which focused on charactering somatic mutations in the cancer genome. One specific aim of these studies is to discover driver genes and mutations that are responsible for tumor initiation, maintenance, progression, and metastasis^1^. However, the process that genome protein-coding alterations are transmitted from the genome to the proteome remains unclear, which has important implications for cancer-genome sequencing studies; because the mutant forms of proteins may be considered as candidate driver mutations/genes and as potential therapeutic targets.

Thus, from multiple omics viewpoints, the processes by which tumor-mutated alleles are transcribed from DNA to mRNA and translated from mRNA to protein must be fully characterized. According to previous studies, the proportions that DNA mutations could be transmitted to RNA products were similar: 36% in triple-negative breast cancer^2^, 41% in non-Hodgkin lymphoma^3^, and 32% in T-cell acute lymphoblastic leukemia^4^. At the protein level, a very low ratio of mutated amino acids was identified in 86 colorectal tumors^5^, in which 796 single amino acid variants were detected. Of these variants, only 64 corresponded to somatic variants reported by TCGA.

Mutated driver genes contain a sufficient number and type of driver gene mutations^6^. Relatively few driver genes/mutations provide selective advantages to tumor cells (i.e., tumor survival and propagation); a tumor typically contains two to eight of ‘driver gene’ mutations^6^. Existing approaches^6^,^7^,^8^,^9^ require a large cohort to identify significantly mutated genes. However, individual tumors of the same type have diverse genomic architectures due to tumor heterogeneity; therefore, there is an urgent need to classify somatic mutations and identify personalized molecular drivers. The challenge remains to discover personalized driver genes in cancers and to assess their impact in a patient-specific manners. It is unknown whether the expressed somatic mutations at the protein level (i.e., the mutant forms of protein) can be used to classify somatic mutations and prioritize personalized candidate driver genes.

In the present study, using a hyper-mutated hepatocellular carcinoma (HCC) patient as a model and deep-sequencing of the patient’s exome, transcriptome and proteome, we aimed to investigate 1) how tumor-mutated alleles are qualitatively and quantitatively transcribed and translated, 2) how mutant allelic fractions are dynamically changed if they are expressed, and 3) the feasibility of classifying somatic mutations and prioritizing personalized candidate mutated driver genes in cancer.

## Results

The expression of tumor-mutated alleles has implications for cancer biology (e.g., identification of cancer driver genes) and clinical practice (e.g., personalized cancer therapy). Here, a hyper-mutated HCC patient was used as a model to characterize the transcriptional and translational patterns of tumor-mutated alleles.

### A hyper-mutated HCC patient

The patient was a 71-year-old man who presented with ‘liver masses’ at the Peking Union Medical College Hospital. The patient had a history of hepatitis B virus (HBV) infection for over 30 years (Table 1). The patient was taking no prescribed medications (e.g., antiviral therapy), and he appeared well. An abdominal ultrasound revealed a large mass in the right lobe of the liver. The histopathological investigation of a specimen from the surgical liver tumor resection revealed HCC. The patient did not have a family history of liver cancer.

By whole-exome sequencing (WES) of the liver cancer and matched cirrhotic tissues (confirmed by histopathological review), we identified 4,998 potential non-silent somatic mutations (i.e., missense, nonsense, nonstop, and translation start site mutations) in this patient (details of WES are presented below). Compared the number of non-silent mutations in the 12 additional HCC tumors sequenced by our group (median number of non-silent mutations, 140), there was an approximate 34-fold increasing in the prevalence of non-silent mutations in the studied patient. The median number of non-synonymous mutations per HCC has been estimated to be 39^6^, which is also significantly less than that observed in the studied patient. The prevalence of somatic mutation corresponded with an exceptionally high number of non-silent mutations in the patient.

**Figure 1:**
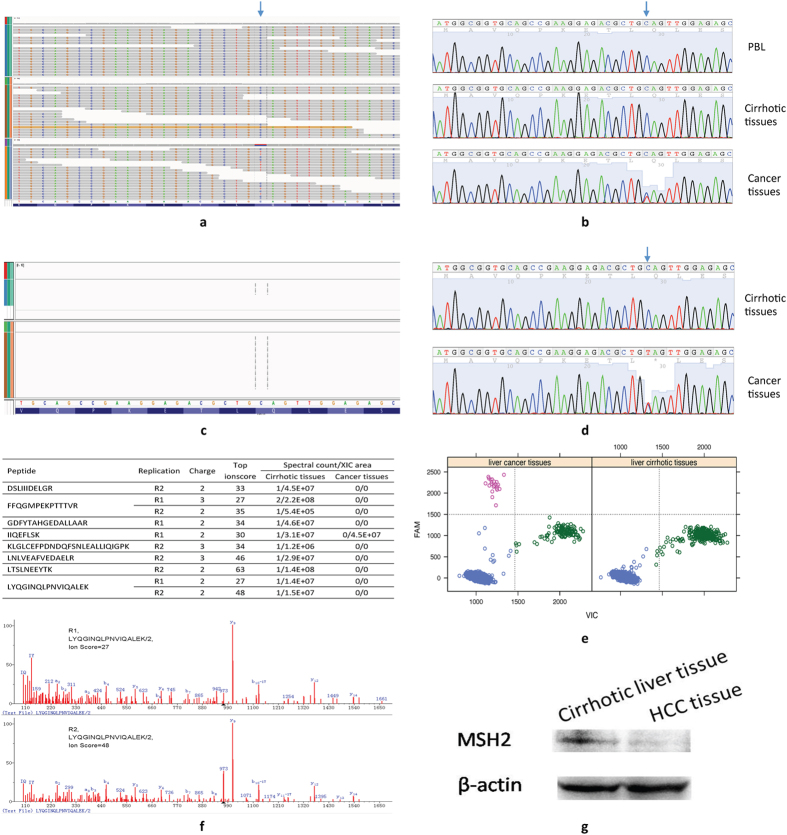
A novel nonsense mutation in *MSH2*. (**a**) WES identified a nonsense mutation, which was confirmed by Sanger sequencing (**b**). The nonsense mutation was not identified by RNA-Seq (**c**), but was confirmed by Sanger sequencing (**d**) and droplet digital PCR (**e**). The arrow indicates the nonsense mutation. (**f**) Mass spectrometry (MS) identified MSH2 peptides in the liver cirrhotic tissue and not in the cancer tissue. The inserted table illustrated the MSH2 peptides identified in the cirrhotic tissue by two independent MS experiments. (**g**) Western plot showing a down-regulated expression of MSH2 in the liver cancer tissue, compared with the cirrhotic tissue. R1, replicate #1; R2, replicate #2; FAM: 5-carboxyfluorescein; and VIC: registered name. PBL, peripheral blood lymphocyte.

DNA mismatch repair corrects mismatches generated during DNA replication and escape proofreading^10^. Tumors with DNA repair defects may contain more mutations than average^11^. Among several non-silent mutations in DNA mismatch repair genes (Supplementary Table S1), a novel heterozygous nonsense mutation in *MSH2* (rs63751099, p.Q10*; chr2:47630358) was noted (Fig. 1a,b) that leads to the early termination of *MSH2* translation. The site was not covered by RNA-Seq (Fig. 1c), but both the wild-type and mutant alleles were transcribed (Fig. 1d,e). Peptides for MSH2 were down-regulated in liver cancer tissue compared with cirrhotic tissue (Fig. 1f,g).

The elevated number of non-silent mutations in the patient provided a rich resource of tumor-mutated alleles and served as a unique model for exploring the transcriptional and translational patterns of tumor-mutated alleles.

### WES, RNA-seq and proteome sequencing

For WES, we captured 185,636 exons of 20,965 genes and sequenced the targeted regions to a mean coverage of 110 × (Supplementary Table S2 and Supplementary Fig. S1a). The cumulative depth of coverage indicated that at least 99% of the bases within the captured regions were covered at least eight times (i.e., depth of coverage ≥ 8) (Supplementary Fig. S1b). In total, 20,382 somatic point mutations were identified and 4,998 of these classified as potential non-silent mutations (Supplementary Fig. S2a)

To reliably and accurately detect variants at the transcriptome and proteome levels, we performed RNA-seq (mRNA_R1 and mRNA_R2) and proteome profiling (pro_R1 and pro_R2) on two replicates of the liver cancer and matched cirrhotic tissues. A summary of the total number and the mapped reads from the RNA-Seq are shown in Supplementary Table S3. Of the 427,455 filtered SNVs in the combined RNA-Seq datasets (mRNA_R1 + mRNA_R2), 13,294 SNVs (3.1%) resided within protein coding regions (refseq): 7,119 (53.6%) were non-synonymous and 5,896 (44.4%) were synonymous (Supplementary Fig. S2b).

For protein identification, the customized protein database constructed from WES and RNA-Seq data was used as the reference database. In the combined datasets (pro_R1 + pro_R2), 1,422,141 spectra were mapped to the sequences (1% FDR at the peptide levels), corresponding to 98,696 unique peptides and 9,255 parsimonious proteins. On average, 153.7 spectra per protein, which is significantly greater than that previously reported for a single liver sample proteomics study, were identified. There were 3,442 identified peptide ions (wide-type or mutant) that covered 1,517 mutation sites (Supplementary Table S4).

The catalog of a large number tumor-mutated alleles compiled by deep-sequencing of the patient’s exome, transcriptome, and proteome allowed for the investigation of the transcriptional and translational patterns of tumor-mutated alleles, i.e., to determine whether and how tumor-mutated alleles were expressed. If a tumor-mutated allele was expressed, whether the wild-type and mutant allele were expressed at the same level could then be determined.

### The characteristics of tumor-mutated allele transcription and translation

At the transcription level, we removed 18 sites where the allelic forms were not reference homozygous in the cirrhotic tissue, thus, 4,980 somatic mutations (in 3,885 genes) were remained. In cases of a transcribed tumor-mutated allele, the allelic forms of the mRNA were required to be heterozygous or alternative homozygous in the liver cancer tissue and to be reference homozygous (or not covered by RNA-Seq) in the cirrhotic tissue.

**Figure 2:**
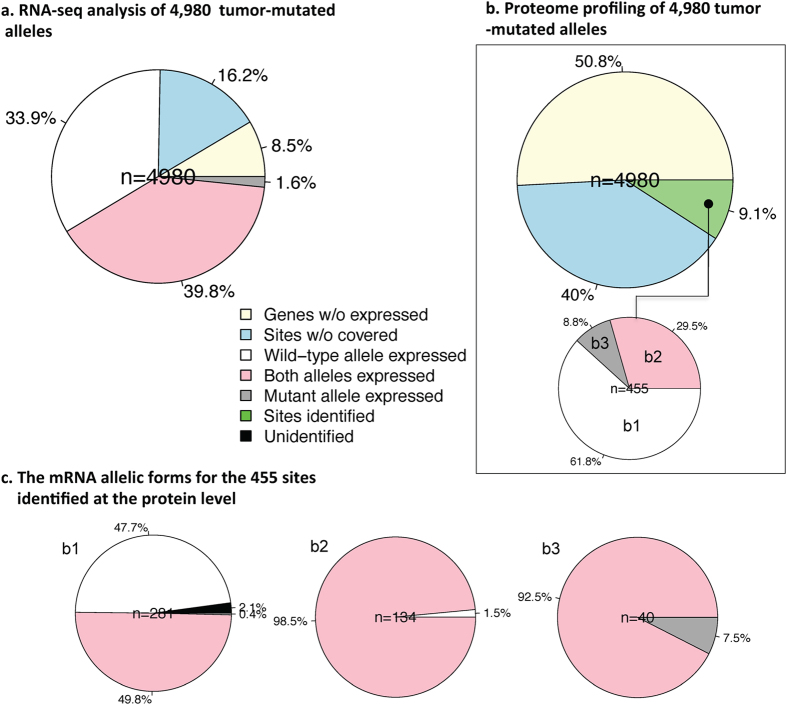
The transcriptional and translational patterns of the tumor-mutated alleles. (**a**) In total, 41.5% of the tumor-mutated alleles (*n* = 2061) were transcribed, and (**b**) 3.5% of the tumor-mutated alleles (*n* = 174) were translated. (**c**) The mRNA allelic forms for the 455 sites identified at the protein level.

Figure 2a illustrates the transcriptional patterns of the tumor-mutated alleles in the combined datasets (mRNA_R1 + mRNA_R2). Notably, 24.7% of the somatic mutations occurred in genes with no observed transcripts (*n* = 1,231, 8.5%) or that were not covered by RNA-Seq reads (*n* = 806, 16.2%). No allelic effect of the tumor-mutated alleles existed if the genes were not expressed in any form. Somatic mutations that were not covered by RNA-Seq may result from low transcripts abundance, which was significantly less than genes where somatic mutations were covered (Kolmogorov–Smirnov test, *p* < 2.2 × 10^−16^). For the remaining sites, only the wild-type allele was transcribed in 33.9% (*n* = 1,688 in 1,461 genes), where both alleles were transcribed in 39.8% (*n* = 1,981 in 1,683 genes), and only the mutant allele was transcribed in 1.6% (*n* = 80 in 79 genes) (Fig. 2a). The patterns were similar in the two independent RNA-Seq replicates (Supplementary Table S5).

Similarly, for the translation analysis (Fig. 2b), we noted that 90.1% of the somatic mutations occurred in genes with no spectrum (*n* = 2,531) or that were not covered by the mass spectrum (*n* = 1,994). We identified 455 sites (9.1%) of mutated amino acids in the liver cancer tissue that not identified in the cirrhotic tissues. Of these sites, only the wild-type alleles were translated in 281 (61.8%), both alleles were translated in 134 (29.5%), and only the mutant allele was translated in 40 (8.8%). The allelic mRNA forms of the 455 sites identified at the protein level are illustrated in Fig. 2c. Of the 455 sites, 68.8% of the tumor-mutated alleles (*n* = 313) were identified by RNA-Seq, and 38.2% (*n* = 174) were identified by proteome profiling. In other words, without considering unexpressed genes and unidentified sites, we found that approximately two-thirds of the tumor-mutated alleles were transcribed, and one-third of the alleles were translated.

### Dynamic changes in mutant allelic fraction

**Figure 3:**
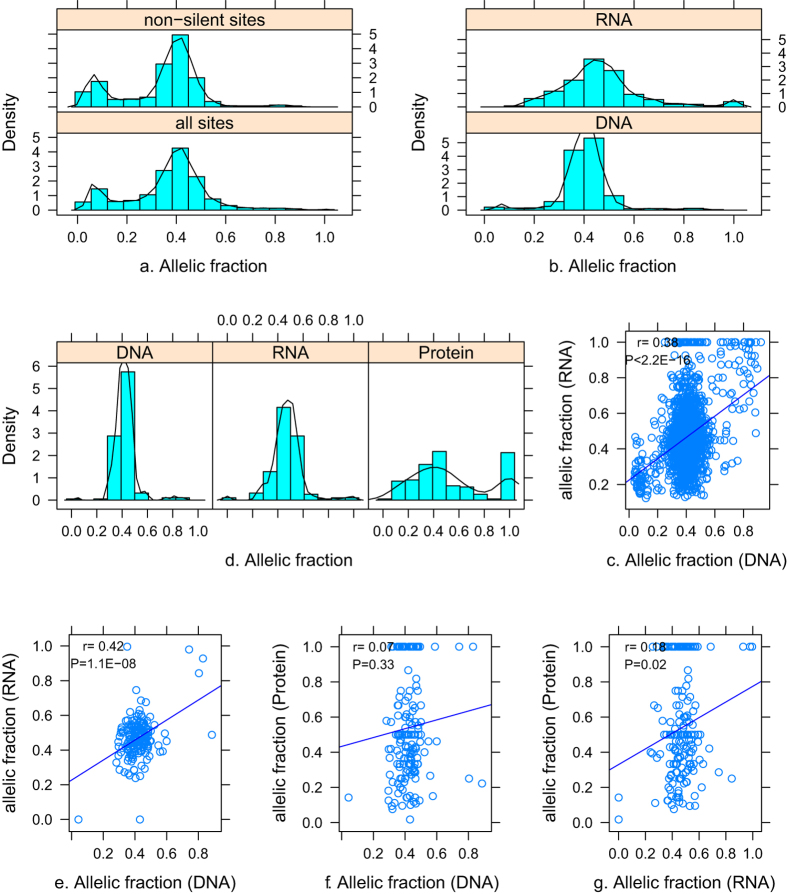
Distribution of the allelic fraction. (**a**) The allelic fraction of all identified somatic mutations (all sites) and non-silent sites identified by WES. The distribution of allelic fraction between all sites and non-synonymous sites differed significantly (Kolmogorov-Smirnov test, *P* < 2.2 × 10^−16^). For the transcribed tumor-mutated alleles, the distribution of the allelic fractions (**b**) and correlation (**c**) between mRNA and genome are shown (*n* = 2061). For translated tumor-mutated alleles, the distribution of the allelic fractions at the protein levels and the corresponding genome and transcriptome (*n* = 174) (**d**), and the correlation in any two of pairwise comparisons (**e–g**). The y-axis in a, b, and d is the probability density of allelic fraction.

Theoretically, a heterozygous founder somatic mutation would be present in virtually all tumor cells with a mutation frequency of 50% in diploid cells. However, a continuous allelic fraction distribution of somatic mutations for tumor heterogeneity was present (Fig. 3a). Obvious dynamic changes of mutant allelic fractions from genome to transcriptome to proteome were noted. The allelic fractions of transcribed tumor-mutated alleles (*n* = 2,061) significantly differed with their corresponding genomic sites (Fig. 3b) (Kolmogorov–Smirnov test, *p* < 2.2 × 10^−16^), and there was a weak but significant correlation between the allelic fractions (*r* = 0.39, *p* < 2.2 × 10^−16^) (Fig. 3c). Similarly, the allelic fractions of translated tumor-mutated alleles (*n* = 174) significantly differed (i.e., with a much wider range) with their counterparts in the transcriptome (*p = *2.0 × 10^−6^) and genome (*p* = 7.7 × 10^−15^) (Fig. 3e). The correlations between allelic fractions in any two of the pairwise comparisons are shown in Fig. 3e–g.

We then ascertained whether the wild-type and mutant allele were expressed at the same level (i.e., allelic-specific expression, ASE) if a tumor-mutated allele was expressed. Of the 2,781 transcribed tumor-mutated alleles, we found a number of 14 genes with a significant bias toward the expression of the mutant allele. Among the 14 sites, only three sites were identified at the protein level, but none exhibited significant ASE. Of the 174 translated tumor-mutated alleles, a missense mutation in *HIST1H4E* (NP_003536, p.D86G) had significant ASE; the tumor-mutated allele was identified at the protein level (allelic fraction = 0.018, total SC = 667) but not at the mRNA levels (total read depth = 69). These results suggested that mutations with ASE at the mRNA level did not necessarily show the corresponding effect at the protein level.

### The transcriptional and translational patterns of tumor-mutated alleles based on allelic fractions

**Figure 4:**
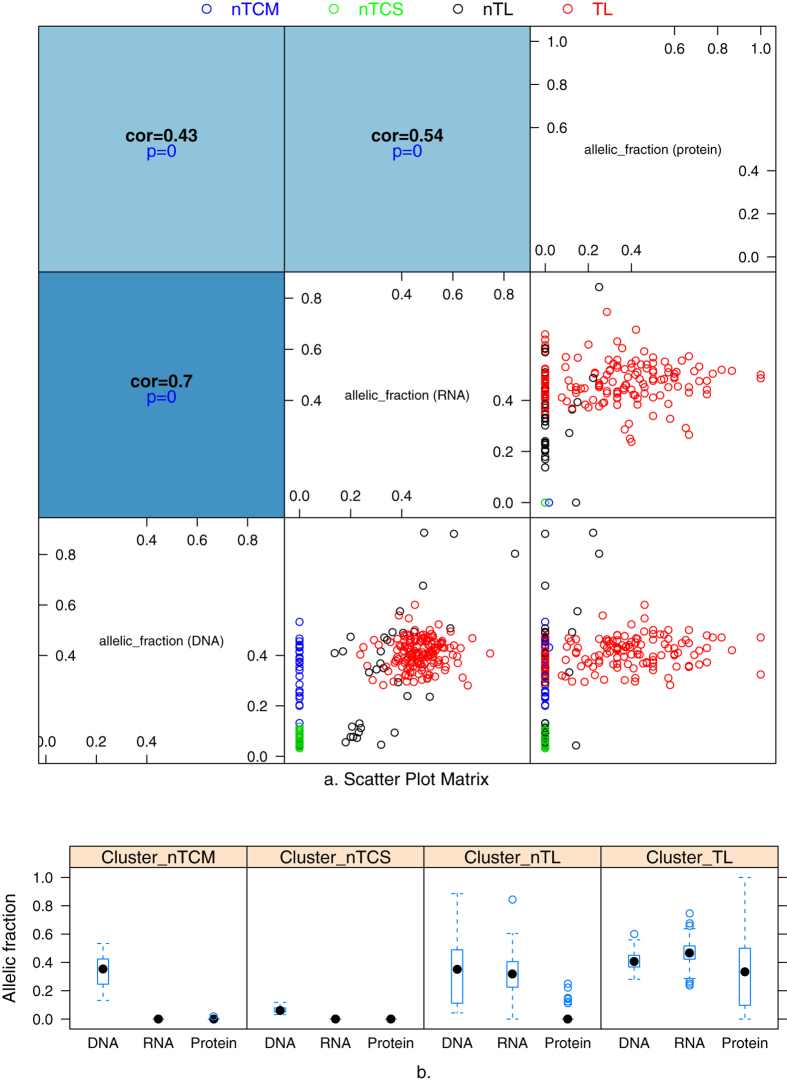
Expression patterns of somatic mutations at the transcriptome and proteome levels. (**a**) A scatter plot matrix indicated four categories of somatic mutations according to the allelic fraction. Different colors represent different clusters. Cluster_nTCM (blue), tumor-mutated alleles with a moderate allelic fraction that were not transcribed; Cluster_nTCS (green), tumor-mutated alleles with a small allelic fraction that were not transcribed; and Cluster_nTL (black), tumor-mutated alleles that were not translated; and Cluster_TL (red), tumor-mutated alleles that were translated. (**b**) Boxplot of allelic fractions at different levels according to clusters.

To avoid the randomization effects, we filtered sites based on a threshold of eight for the site depth in WES or RNA-Seq (DP ≥ 8), and of three for the spectral count (SC ≥ 3) in MS. After filtration, 257 (in 237 genes) of 455 sites remained for cluster analysis (i.e., grouped the somatic mutations according to their expression). Figure 4a presents scatterplot matrix (SPLOM) plots of all combinations of allelic fractions at each level (genome, transcriptome and proteome). In total, 10.5% (*n* = 27) of the tumor-mutated alleles with moderate allelic fractions (0.13–0.53, blue dots; Cluster_nTCM, tumor-mutated alleles not transcribed with a moderate allelic fraction) and 17.5% (*n* = 45) with small allelic fractions (0.03–0.11, green dots; Cluster_nTCS) were not transcribed. Furthermore, 12.8% (*n* = 33) of these alleles were transcribed but not translated (black dots; Cluster_nTL, tumor-mutated alleles not translated). The remaining 59.1% (*n* = 152) of the tumor-mutated alleles were present at the protein level (red dots; Cluster_TL). The correlation between the allelic fractions at any two of levels, gradually decreased from genome to transcriptome to proteome, as expected. Box plots of allelic fractions of the four clusters at the different levels (Fig. 4b) indicated that subsets of tumor-mutated alleles were eliminated at transcriptional and translational levels.

### Effects of tumor-mutated alleles on protein expression

Tumor-mutated alleles affect gene expression at both the transcriptional and translational stage^5^. To determine the effects of translational and/or post-translational regulation after correcting for the transcriptional effects, we defined the measure of *DPRE* as the difference in the normalized fold change (FC) of protein and RNA expression between liver cancer and cirrhotic tissues. The *DPRE* only measures the relative protein abundance after correcting for variations in RNA abundance. A *DPRE* > 0 suggested relatively high protein abundance, and a *DPRE* < 0 suggested low expression.

**Figure 5:**
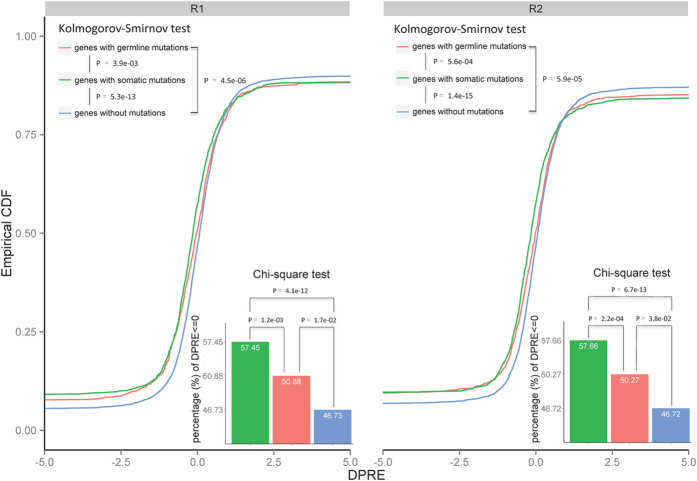
Empirical cumulative density of the *DPRE* in genes within three groups: with somatic mutations, with germline variations, and without mutations. R1 and R2 are the two independent MS experiments, and the two combined RNA-Seq data sets were used to calculate DPRE. The Kolmogorov–Smirnov test was performed to test whether the distribution of any two of the groups significantly differed. The inserted bar chart illustrated the percentage of genes with DPRE ≤ 0 in the three groups.

We compared the *DPRE* values of genes grouped as somatic mutations, germline variations, and without mutations. For clarification, the expression level of a given gene (i.e., FC) was required to be consistent in the liver cancer and cirrhotic tissues in the two RNA-Seq replicates. We noted that the *DPRE* of genes with somatic mutations (*n* = 3,351) was significantly less than that of genes with germline variations (*n* = 3,207) (Pro_R1: *p* = 3.9 × 10^−3^, and Pro_R2: *p* = 5.6 × 10^−4^, Kolmogorov-Smirnov test) and genes without mutations (*n* = 11,822) (Pro_R1: *p* = 5.3 × 10^−13^, and Pro_R2: *p* = 1.4 × 10^−15^) (Fig. 5). Additionally, the percentage of genes with somatic mutations with *DPRE* ≤ 0 (58%) was significantly greater than that for genes with germline variations (51%) (Pro_R1: *p* = 1.2 × 10^−3^, and Pro_R2: *p* = 1.7 × 10^−2^; χ^2^ test) and without mutations (47%) (Pro_R1: *p* = 2.2 × 10^−4^, and Pro_R2: *p* = 3.8 × 10^−2^). These results indicated that tumor-mutated alleles could significantly reduce the protein abundance via translational and/or post-translational regulation, especially for genes with somatic mutations.

To investigate the possible mechanisms of expression insufficiency for proteins with somatic mutations, we analyzed the distribution of *DPRE* for the four clusters shown in Figure 4a. We found that Cluster_nTL has the highest percentage of *DPRE* ≤ 0 (69.6%), whereas Cluster_nTCM has the lowest percentage (57.1%), suggesting that the protein expression insufficiency was partially caused by the elimination of tumor-mutated alleles. However, we cannot exclude other potential mechanisms (e.g., the percentage of *DPRE* ≤ 0 was >50% in the Cluster_nTCS). We evaluated the possible effects of somatic mutations in Cluster_nTL and Cluster_TL on protein stability^12^. On average, somatic mutations in Cluster_nTL destabilized protein more likely (mean ΔΔG value = −0.94) than that in Cluster_TL (mean ΔΔG value = −0.81).

### Classification of somatic mutations in cancer: prioritizing personalized candidate mutated driver genes

The classification of somatic mutations, i.e., identification of potential mutated driver genes, especially in a patient-specific manner, is of interest. We proposed a gene-selection strategy to classify somatic mutations and prioritize personalized mutated driver genes by incorporating the expression patterns of tumor-mutated alleles and known cancer driver genes as well as by conducting functional network analysis. We hypothesized that a personalized candidate mutated driver gene would follow these criteria: 1) the tumor-mutated allele would be expressed at the protein level; 2) the allelic fraction of an expressed tumor-mutated allele would be comparable at the DNA, mRNA, and protein levels; and 3) the given gene would be associated with the liver function and disease (e.g., liver cancer), as extracted from Ingenuity Pathway Analysis (IPA).

**Figure 6:**
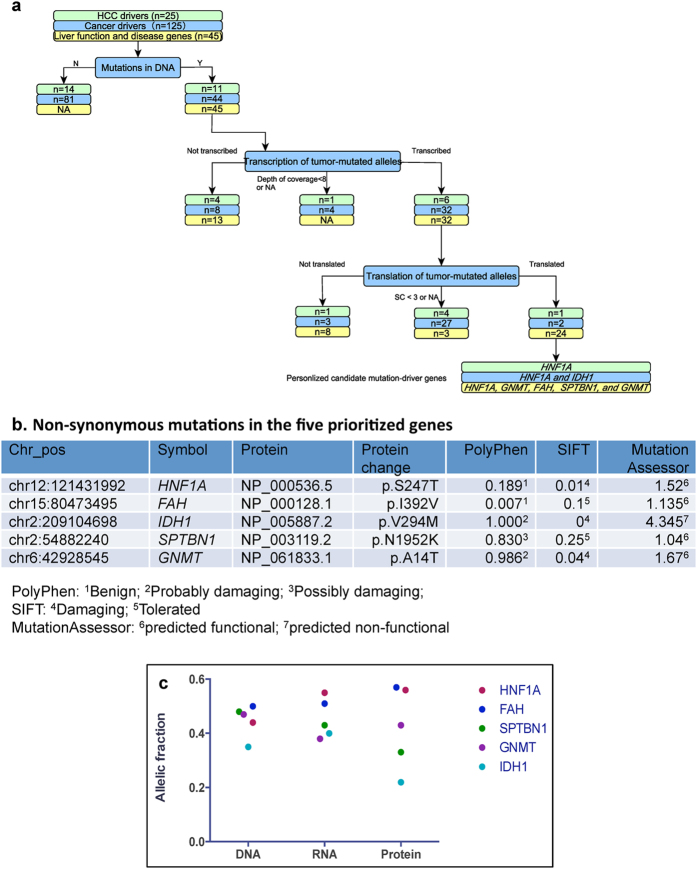
The classification of somatic mutations and prioritization of personalized candidate mutated driver genes. (**a**) The transcription and translation of tumor-mutated alleles in 25 potential HCC driver genes^13^, 125 cancer driver genes^6^, and 45 genes associated with liver function and diseases extracted from IPA. (**b**) Non-synonymous mutations in the five prioritized genes and their predicted functional impact on protein sequence or structure. Probably damaging: it is with high confidence supposed to affect protein function or structure; Possible damaging: it is supposed to affect protein function or structure; (**c**) The allelic fractions of five personalized candidate mutated driver genes at the genome, transcriptome, and proteome levels.

We first investigated the expression of tumor-mutated alleles in known cancer driver genes that were summarized in two elegant review articles^6^,^13^. Figure 6a summarized how tumor-mutated alleles of known cancer drivers were identified at the DNA, mRNA and protein. For example, of the 25 candidate driver genes in HCC with recurrent genetic alterations^13^, somatic mutations were identified in 11 sites (11 genes). Of these 11 sites, six tumor-mutated alleles of the 10 identified sites (depth of coverage ≥ 8) were transcribed (Supplementary Table S6). Of three sites identified at the protein level, only the mutated allele (p.S247T, NP_000536.5) of *HNF1A* was translated in addition to the wild-type allele (Supplementary Table S6). Therefore, *HNF1A* was prioritized as a personalized candidate mutated driver gene. Similarly, of the 125 previously summarized mutated driver genes^6^, *HNF1A* and *IDH1* were prioritized (Supplementary Table S7).

We cannot exclude the possibility that other mutant forms of proteins may drive the hepatocarcinogenesis. Therefore, we analyzed 237 genes used for clustering and incorporated functional pathway analysis. At least 10 pathways (45 genes) extracted from the network constructed by Ingenuity^®^ Pathway Analysis (IPA, http://www.ingenuity.com/products/ipa) were significantly associated with liver function and disease (e.g., liver cancer) (Supplementary Fig. S3a). In the studied patient, of 66 somatic mutations in the 45 genes in these pathways, 42 mutated alleles in 32 genes were transcribed, and 26 mutated alleles in 24 genes (54 identified sites) were translated (Fig. 6a and Supplementary Table S8). Of the 24 genes, four genes (*HNF1A*, *GNMT*, *FAH*, and *SPTBN1*) were causally related to the occurrence of liver cancer (Table 2 and Supplementary Fig. S3b).

In total, five genes (*HNF1A*, *IDH1*, *FAH*, *GNMT*, and *SPTBN1*) were prioritized as personalized candidate mutated driver genes in the studied patient. These genes were involved in important known cancer signaling pathways (e.g., chromatic modification). *HNF1A* and *IDH1* regulate the core cellular process of cell fate. *HNF1A* has a potent inhibitory effect on HCC^14^, and *HNF1A* mutation are associated with both benign and malignant primary liver cell tumors^15^. *IDH1* mutations have stimulated the burgeoning field of tumor metabolism^16^; these mutations impair histone demethylation and results in a block to cell differentiation^17^. *Fah* homozygous mutant mice have an increased incidence of HCC^18^,^19^. GNMT regulates HCC growth in part by modulating mTOR/raptor signaling pathway^20^,^21^. *Gnmt* homozygous mutant mice have an increased HCC incidence^22^. Disrupting TGF-β signaling through SPTBN1 leads to HCC via cyclin D1 activation^23^, and *Sptbn1* heterozygous mutant mice have an increased HCC incidence^24^,^25^. Of the five non-synonymous mutations, four were predicted to be damaging by PolyPhen, SIFT or MutationAssessor (Fig. 6b). The allelic fractions of the tumor-mutated alleles in these five genes indicated that the mutant forms of proteins were expressed at a moderate proportion (Fig. 6c).

### Validation of translated tumor-mutated alleles by selected reaction monitoring (SRM)

We selected 14 translated tumor-mutated alleles for validation using SRM (Supplementary Table S9), including three genes (i.e., *HNF1A, FAH* and *SPTBN1*) that have been causally related to liver cancer. The fractions from which the mutated and wild-type alleles were identified were subjected to SRM analysis on a triple quadruple mass spectrometer. At least eight transitions were required (the Skyline software) for each SRM measurement. The results showed confident validation for most of the targeted peptides. The SRM profiles of the peptides containing the mutated and wild-type amino acid corresponding to the mutation in *HNF1A* (chr12:121431992, NP_000536, p.S247T) are shown in Supplementary Fig. S4.

## Discussions

In the present study, using a hyper-mutated HCC patient as a model, we qualitatively and quantitatively studied the transmission of tumor-mutated alleles from DNA to RNA to protein, i.e., the transcriptional and translational pattern of tumor-mutated alleles. Our results provided the direct evidence that tumor-mutated alleles can be eliminated by translational or post-translational mechanisms, and 42% and 3.5% of the tumor-mutated alleles were identified to be transcribed and translated, respectively. The transcriptional and translational patterns of tumor-mutated alleles may enable the prioritization of personalized candidate mutation-driver genes.

Tumor-mutated alleles were not always expressed as a result of various molecular mechanisms. First, tumor-mutated alleles may reside in genes that are not expressed. Second, transcription-coupled repair (TCR)^26^ restores lesions from the transcribed strands of actively transcribed genes faster than from non-transcribed strands. Similar TCR mechanism on preferentially somatic mutations has been reported previously^27^,^28^. Approximately 34% of the somatic mutations were not transcribed due to its allelic effects on mRNA in the studied patient. Third, by reducing translational efficiency^29^ or protein stability, somatic mutations may negatively impact protein abundance.

The transcriptional and translational patterns of tumor-mutated alleles may enable the classification of somatic mutations, i.e., prioritization of personalized mutated driver genes. The proposed gene-selection strategy (i.e., tumor-mutated allele expression-based) focused on patient-specific mutations regardless of the mutation frequency, wherein genes with expressed tumor-mutated alleles are more likely to be served as candidate drug targets for personalized medicine. The strategy builds upon the following rationales: 1) near-saturation of the number of frequently altered mutated driver cancer genes occurs^6^,^30^; 2) the effect of somatic mutations can be determined by expression at the mRNA and/or protein levels; and 3) the genes have been implicated to be causally related to cancer development.

We did not identify somatic mutations in *TP53* or *CTNNB1* (the average depth of coverage was 55 and 134, respectively), which are frequently mutated (~20%) in HCC^31^, in the studied patient. Although a non-synonymous mutation (chr1 27056157; NP_006006, p.D385N) in *ARID1A* (mutated in 10–16% of HCCs) was identified, the tumor-mutated allele was not transcribed. Our identification may help classify somatic mutations based on its expression patterns and indicate the personalized mutated driver genes leading to hepatocarcinogenesis in the patient. The novel nonsense mutation in *MSH2* was an initiation hit (or a ‘trigger’) (as posited in the mutator phenotype hypothesis^32^), leading to a greatly increased rate of genome-wide point mutations^33^. Using our gene-selection strategy, we selected and prioritized five personalized candidate mutated driver genes that may contribute to hepatocarcinogenesis promotion and progression. The moderate mutant allelic fraction (0.22–0.57) may have affected the encoded-protein structure and function. Additionally, prediction of the consequences of non-synonymous mutations in these five genes suggested that these mutations had damaging effects on the protein structure and function.

There are several limitations in the present study. A major limitation is the lack of functional validation of the inferred driver genes, and follow-up functional experiments could strengthen the predictions. However, previous studies have suggested that the inferred genes were causally related to liver cancer (Table 2). Second, multiple patients are needed to demonstrate the generalizability of the proposed method. Third, the mutant allelic fraction requires validation, e.g., by SRM at the protein level^34^. We validated the nonsense mutation in *MSH2* by ddPCR and the mutated peptides in *HNF1A*, *SPTBN1*, and *FAH* by SRM. The measure of *DPRE* could be used to prioritize personalized mutated drivers. However, we cannot integrate such a measure in the model due to the lack of training data in the present study. The inherent deficiencies in current proteome technologies (e.g., the relatively low sequence coverage) led to a low percentage of identification of tumor-mutated alleles. In this study, we tried to bypass the technological limitations (e.g., sequencing depth and bias) to obtain the reliable results. Of the 455 sites identified, MS identified 281 wild-type alleles and 40 mutated alleles. Suppose all wild-type alleles could be expressed, the 40 mutated alleles should miss the identification of their wild-type alleles. So, at least there are 241 alleles (281-40) whose mutated alleles were not expressed. The significant bias indicated that tumor-mutated alleles tending not to be expressed are reliable despite the limitations of proteome technologies. It should also be noted that synonymous mutations are very important in cancer, which is relevant to translation control and tumorigenicity^35^.

In conclusion, using a hyper-mutated HCC patient as a model, we found that only a small proportion of the tumor-mutated alleles identified at the DNA level were transcribed and translated. As a proof-of-concept, leveraging a multi-omics strategy based on the expression patterns of tumor-mutated alleles may enable the classification of somatic mutations and prioritization of personalized candidate drivers. Furthermore, identifying the mutated amino acids linked to genomic somatic mutations may be beneficial for tumor reclassification and personalized cancer therapy.

## Materials and Methods

### Whole exome sequencing (WES) and transcriptome sequencing (RNA-Seq)

A 71-year-old male with hepatitis-B virus (HBV) associated hepatocellular carcinoma (HCC) was seen at the Peking Union Medical College Hospital. The patient provided the written informed consent and the Institutional Review Board (IRB) office at the Second Affiliated Hospital of Chongqing Medical University approved the study. All experiments were performed in accordance with relevant guidelines and regulations. We performed WES on the liver cancer tissue, matched cirrhotic tissue (all tissues after surgical resection were frozen at liquid nitrogen until genomic DNA and total RNA extraction), and whole blood banked from this patient. Two independent RNA-Seq experiments were performed on the liver cancer and matched cirrhotic tissues. A detailed description of the WES and RNA-Seq are provided in the Supplementary materials.

We analyzed WES reads using a pipeline of integrated multiple bioinformatics tools (Supplementary Fig. S5). We used MuTect^36^ for the reliable and accurate identification of somatic point mutations by comparing the liver cancer and cirrhotic tissues. Oncotator (www.broadinstitute.org/cancer/cga/oncotator) was used to annotate the somatic mutations. The reliable identification of genomic variants from RNA-Seq data remains a challenge due to the complexity of the transcriptome. Piskol *et al*.^37^ presented a highly accurate approach to identify single-nucleotide variants (SNVs) in RNA-Seq data, which was modified to identify sequence variants from our RNA-Seq data (Supplementary Fig. S6).

### Sanger sequencing, Droplet digital PCR (ddPCR), and Western blot

Traditional Sanger sequencing was performed using BigDye terminator chemistries on an ABI 3730xl (Life Technologies®; Carlsbad, CA) sequencer. ddPCR for the nonsense mutation in *MSH2* was performed using a Bio-Rad^®^ QX100 system. Details for ddPCR and Western blot are available in the Supplementary materials.

### Proteome profiling by mass spectrometry (MS)

A detailed description of the in-depth proteome analysis of the liver tissue samples, including protein extraction and in-solution digestion, serial peptide prefractionation by isoelectric focusing (IEF) and high pH reversed-phase chromatography, as well as mass spectrometric analysis of the peptide mixture is provided in the Supplementary materials.

### Construction of a customized single amino acid variant (SAV) database and MS database searching

To increase the power in identifying SAVs at the protein level, we constructed customized SAVs database based on matched WES and RNA-Seq data, including germline variations and somatic mutations. We included all mutations from the liver cancer and cirrhotic tissues for MS database searching. Database searching [RefSeq + customized RNA-Seq-based mutation sequences + contamination proteins (115 proteins, ftp.thegpm.org/fasta/cRAP)] was performed by Mascot for the raw mass spectra files (Supplementary materials). The cross-search between the liver cancer and cirrhosis tissues was used to obtain paired quantification results. The identified peptides were mapped to the chromosome locations.

### Estimation of allelic fraction

To quantify the relative amount of a mutant allele in a heterogeneous tumor samples, we used the ‘allelic fraction,’ which is defined as the number of times a mutated base is observed divided by the total number of times any base is observed at the locus^38^,^39^. Similarly, we defined the allelic fraction at the protein level based on the SC of the mutation site. We used the expectation-maximization algorithm (the ‘mclust’ package in *R*, www.r-project.org) to classify allelic fractions of DNA, mRNA, and protein into groups.

To test for a bias toward the expression of the wild-type or the mutant allele, we applied Fisher’s exact test between the total number of reads and the depth of coverage supporting the wild-type allele in the WES, RNA-seq and MS data^40^. Cases with a false discovery rate (FDR) < 0.05 were considered significant.

### Effects of translational and post-translational regulation on the expression of mutated genes

We estimated the levels of gene expression using ‘cufflinks’^41^ (i.e., FPKM, reads per kilobase of exon model per million mapped reads^42^). The protein abundance was estimated by XIC for two replicates (R1 and R2) separately. The logarithm of fold change (FC) was used to measure differential gene expression between the liver cancer tissue (*Exp*_*cancer*_) and cirrhotic tissue (*Exp*_*cirrhosis*_), i.e., *FC* = *log*_2_(*Exp*_*cancer*_/*Exp*_*cirrhosis*_). To include all data in the analysis, we adjusted the values of 0 and infinity in the division. That is, if *Exp*_*cirrhosis*_ = 0, we defined *FC* = 10; and if *Exp*_*cancer*_ = 0, *FC* = −10.

To investigate the effects of translational and post-translational regulation, we corrected for the effects of transcriptional regulation on the mRNA levels. We first normalized the FC for transcripts and proteins separately, and then defined a measure (*DPRE*, Difference of normalized fold change of Protein and RNA Expression) to quantify the difference of the normalized protein and mRNA expression levels as:

*DPRE* = Protein(*Normalized FC*)-mRNA(*Normalized FC*), where

*Normalized FC* = [*FC*-median(*FC*)]/[*Q*_3_(*FC*)-*Q*_3_(*FC*)], and *Q3*(*FC*) and *Q*_1_(*FC*) are the upper and lower quantile of the *FC*, respectively.

### Variants selected for validation by selected reaction monitoring (SRM)

We selected 14 sites which tumor-mutated alleles were identified at the protein levels for further validation by SRM (Supplementary Table S9). Detailed methods are available in the Supplementary Materials.

## Additional Information

**How to cite this article**: Ding, K. *et al*. Leveraging a Multi-Omics Strategy for Prioritizing Personalized Candidate Mutation-Driver Genes: A Proof-of-Concept Study. *Sci. Rep*. **5**, 17564; doi: 10.1038/srep17564 (2015).

## Supplementary Material

Supplementary Information

## Figures and Tables

**Table 1:** Clinical characteristics of the patient.

	Patient	Reference range
Age (years)	71 y	–
HBsAg	(+) >250 IU/ml	(−) <0.05 IU/ml
HBsAb	(−) 0.21 mIU/ml	(−) <10.0 mIU/ml
HBeAg	(−) 0.24 S/CO	(−) <1 S/CO
HBeAb	(+) 0.01 S/CO	(−) >1 S/CO
HBcAb	(+) 11.62 S/CO	(−) <1 S/CO
HBV DNA	<10^3^ copies/ml	<10^3^ copies/ml
HCV Ab	(−) 0.07 S/CO	(−) <1 S/CO
AFP	3.6 ng/ml	0.00–13.20 ng/ml
CEA	2.11 ng/ml	0.00–5.50 ng/ml

HBsAg, Hepatitis B surface antigen; HBsAb, Antibody to hepatitis B surface antigen; HBeAg, Hepatitis B e antigen; HBeAb, Antibody to hepatitis B e antigen; HBcAb, Hepatitis B core antibody; HCV, Hepatitis C virus; AFP, Alpha-fetoprotein; CEA, Carcinoembryonic antigen; and S/CO, Sample/cut off.

**Table 2:** Genes causally related to liver cancer with mutated alleles expressed at the protein level.

Symbol	Effects	Reference
*HNF1A*	Down regulation of human *HNF1A* mRNA in HCC is associated with HCC in human	43
	An potent inhibitive effect of *HNF1α* on HCC by inducing the differentiation of hepatoma cells into mature hepatocytes and G(2)/M arrest	14
*FAH*	Homozygous mutant mouse *Fah* increases incidence of HCC in mouse	18,19
*GNMT*	GNMT is involved in growth of HCC	20
	Homozygous mutant mouse *Gnmt* increases incidence of HCC	22
*SPTBN1*	Heterozygous mutant mouse *Sptbn1* gene increases incidence of HCC in mouse	24,25

HCC, hepatocellular carcinoma.
