# Supplementary material for: Leveraging a Multi-Omics Strategy for Prioritizing Personalized Candidate Mutation-Driver Genes: A Proof-of-Concept Study
Source: Sci Rep. 2015 Dec 3;5:17564. doi: 10.1038/srep17564 (PMC4668376; doi:10.1038/srep17564)
Supplement: Supplementary Information [file srep17564-s1.pdf]

Leveraging a Multi-Omics Strategy for Prioritizing Personalized Candidate Mutation-Driver Genes: A Proof-of-Concept Study

Keyue Ding[1,4,*], Songfeng Wu[2,4], Wantao Ying[2,4], Qi Pan[1], Xiaoyuan Li[3], Dachun Zhao[4], Xianyu Li[2], Qing Zhao[2], Yunping Zhu[2], Hong Ren[1,*], and Xiaohong Qian[2,*]

1. Institute for Viral Hepatitis, Key Laboratory of Molecular Biology for Infectious Diseases, Ministry of Education of China; The Second Affiliated Hospital of Chongqing Medical University, Chongqing, P. R. China, 400010
2. State Key Laboratory of Proteomics, National Protein Science Beijing Center, Beijing Proteome Research Center, Beijing Institute of Radiation Medicine, Beijing, P.R. China, 102206
3. Department of Medical Oncology, Peking Union Medical College Hospital, Chinese Academy of Medical Sciences and Peking Union Medical College, Beijing, P. R. China, 100730
4. Department of Pathology, Peking Union Medical College Hospital, Chinese Academy of Medical Sciences and Peking Union Medical College, Beijing, P. R. China, 100730
5. These authors contributed equally to this work

*, co-corresponding authors

Supplementary Materials

Library construction, exome capture, and whole exome sequencing (*WES*)

The qualified genomic DNA sample was randomly fragmented into fragments with a base pair peak of 150 to 200bp, and then adapters were ligated to both ends of the resulting fragments. The adapter-ligated templates were purified by the AgencourtAMPure SPRI beads and fragments with insert size about 200bp were excised. Extracted DNA was amplified by ligation-mediated (LM-) PCR, purified, and hybridized to the SureSelect Biotinylated RNA Library (BAITS) for enrichment. Hybridized fragments were bound to the streptavidin beads whereas non-hybridized fragments were washed out after 24 hours. Captured LM-PCR products were subjected to Agilent 2100 Bioanalyzer to estimate the magnitude of enrichment. Each captured library was then loaded on Hiseq2000 platform. We performed high-throughput sequencing for each captured library independently to ensure that each sample met the desired average fold-coverage (i.e., at least 100×). Raw image files were processed by Illumina base calling Software (version 1.7) for base calling with default parameters and the sequences of each individual were generated as 90bp paired-end reads.

RNA-Seq

After the total RNA extraction and DNase I treatment, magnetic beads with Oligo (dT) are used to isolate mRNA from the total RNA. The mRNA was fragmented into short fragments mixed with the fragmentation buffer. Then, cDNA was synthesized using the mRNA fragments as templates. Short fragments were purified and resolved with EB buffer for end preparation and adenine addition. The short fragments were then connected with adapters. The suitable fragments were selected for PCR amplification. The quality of the sample library was assessed by Agilent 2100 Bioanalyzer and ABI StepOnePlus Real-Time PCR system. Finally, the cDNA library was sequenced using Illumina HiSeq™ 2000 and 90 bp paired-end sequences were generated.

ddPCR validation for the nonsense mutation in MSH2

The ddPCR reaction mixture consisted of 10 μl of a ddPCR master mix (Bio-Rad®), 0.8 μl of MSH2 primers mix (final Concentration: 900nM), 1μl MGB probes mix (Invitrogen®, final concentration: 500nM), and 2μl of sample solution in a final volume of 20 μl. MGB probes were obtained as a custom design from Life Technologies: 5'-FAM-AGGAGACGCTGTAGTT-MGB-3' (mutant allele), and 5'-VIC-AAGGAGACGCTGCAGT-MGB-3' (wild-type allele).

Western blot of MSH2

Frozen tissues were homogenized using the Tissue Homogenizer (Omni, American) in ice-cold RIPA lysis buffer (50mM Tris base, pH 7.4, 150mM NaCl, 1mM EDTA, 1% Triton X-100, 1% sodium deoxycholate, 0.1% SDS, 10nM PMSF) containing protease inhibitor cocktail tablets (Roche, Switzerland). Then, the tissues were lysed at 4°C for 30min in lysis buffer and the lysates were clarified by centrifugation at 15,000rpm at 4°Cfor 30min. Protein concentration was determined using the 2D Quantification Kit (Amersham Biosciences, Sweden). The protein samples (50ug in each well) were separated by SDS–PAGE and transferred to PVDF membrane. The membranes were blocked with 5% non-fat dry milk in TBS-T buffer (20mM Tris, pH 7.6, 100mM NaCl, 0.5% Tween-20) overnight at 4°C, followed by 3 hours of incubation with the primary antibody (Santa Cruz, American, 1:100 dilution) in TBS-T buffer containing 5% non-fat dry milk at room temperature. After washing three times with TBS-T buffer, the membranes were incubated with an HRP-conjugated goat anti-mouse IgG as the secondary antibody (MULTISciences, China, 1:5000 dilution) for 1 hour at room temperature. The membranes were then washed three times in TBS-T buffer and the reactions were visualized with the ECL detection system. All of the analyses were repeated at least three times.

Protein extraction and in-solution digestion

The liver tissues were resuspended in ice-cold lysis buffer (8 M urea, 20 mM Tris-HCl, pH 8.0, 1mM Na2VO3; 5mM NaF; 20mM DTT, 1% protease inhibitor cocktail). Acid cleaned glass beads were added and the tissue protein was extracted by collision for 30s with 70Hz energy. The protein extraction were moved to a new tube and further sonicated for 200 W × 1 s (working) × 2 s (resting) × 30 circles. The suspension was centrifuged at 12000 g for 15 min at 4 °C. Protein concentration was determined by a BCA assay.

For in-solution digestion, the tissue lysates were first reduced with 10mM DTT for 4h at 37 °C and alkylated with 40mM IAA in dark for 1 hour. Excess IAA was quenched by adding 20mM of DTT. The urea concentration in the sample solution was reduced to 1M with 50 mM NH4HCO3, and proteins were digested with trypsin (Promega, USA) overnight. The protein to enzyme ratio was 100:1 and protein digestion was stopped by adding formic acid at 0.1% final concentration.

Serial peptide prefractionation by IEF and high pH reversed-phase chromatography

Tryptic peptides were fractionated according to the manufacturer’s protocol using ImmobilinTM DryStrip, pH 3-10, 13cm (GE Healthcare) on an OFFGEL 3100 system (Agilent Technologies, USA). Twelve fractions were collected from the fractionator and then every three

continuous fractions were concatenated into one faction, which resulted into 4 fractions from the IEF separation.

For the following high pH reversed-phase chromatography (bRP) separation, an L-3000 HPLC system (Rigol) by using a RP column (5 μm, 300 Å, 250 mm × 4.6 mmI.D., Waters). Mobile phases A (2% acetonitrile in water (v/v), adjusted pH to 10.0 using NH3·H20) and B (98% acetonitrile in water (v/v), adjusted pH to 10.0 using NH3·H20) were used to develop a 40 min gradient. The flow rate was 0.7 mL/min and the column oven was set as 45°C. Eluent was collected every minute. Then the fractions were dried under vacuum (Thermo Savant). Before MS identification, the peptides were reconstituted in 0.1% (v/v) FA, 2% (v/v) acetonitrile in water, and pooled in a discontinuous mode into 24 (for the acid end IEF fraction) or 12 fractions (for the other three IEF fractions).

Mass spectrometric analysis of peptide mixture

Peptide mixture was measured on an Q-Exactive mass spectrometer (Thermo Fisher Scientific) equipped with an Easy-nLC nanoflow LC system (Thermo Fisher Scientific). Peptides were separated on a C18 column (3 μm, 100 Å, 75 um ID ×10 cm). Mobile phase A consisted of 0.1% FA, 2% acetonitrile in water, and mobile phase B consisted of 0.1% FA, 98% acetonitrile in water. The solvent gradient was set as follows: 5%-8% B, 3 min; 8%-22% B, 30min; 22%-32% B, 5 min; 32%-90% B, 1 min; 90% B, 6 min. For the Q-Exactive part, the source was operated at 2.2 kV. For full MS survey scan, AGC target was 3e6, scan range was from m/z 300 to 1400 with the resolution of 70,000. The 75 most intense peaks with charge state 2 and above were selected for sequencing and fragmented in the ion trap by HCD with normalized collision energy of 27%. Exclude isotope item was on and dynamic exclusion time was set as 18s.

Selective reaction monitoring (SRM) validation

SRM transitions were calculated using Skyline software (https://skyline.gs.washington.edu) from the peptide amino acid sequence. At least the most intensity 8 fragment ions/each peptide were selected to setup MRM transitions. The MRM validation experiment was performed on Eksigent nanoLC-Ultra® 2D System and QTRAP 6500 system (AB SCIEX). And the MRM peaks were extracted and analyzed with Skyline software. The peptide with at least 8 MRM peaks was considered to be confirmed by MRM experiment.

For the mass spectrometric analysis, about 2 microgram peptide mixture was separated on an Eksigent nanoLC-Ultra® 2D System with a cHiPLC®-nanoflex system (Eksigent, USA) in trap elute mode. In each injection, the sample was desalted on a 200 μm x 6 mm trap chip and then

eluted onto a 200 μm x 150 mm column chip for MS analysis. The media for both the trap and column chips were ChromXP C18-CL (3μm, 120Å, Eksigent). Peptides were separated using a linear gradient formed by A (2% ACN, 0.1% FA) and B (98% ACN, 0.1% FA) from 7–35% of B over 75 minutes at a flow of 300nL/min.

The MS analysis was performed on a QTRAP® 6500 system (AB SCIEX). The optimal acquisition parameters were as follows: curtain gas (30), ionspray voltage (2300V), ion source gas (15), interface heater temperature (150℃), collision gas (High), declustering potential (80), entrance potential (10) and collision cell exit potential (15). The resolution parameters of the first and the third quadrupole were set as "unit". The target ions were transmitted with a narrow window (0.7 Da). The dwell time was 20 ms for every transition.

MS database searching

All raw files of mass spectra were converted into mzXML and MGF files using the msconvert module in the Trans-Proteomic Pipeline (TPP v4.5.2). The MS/MS peak lists were searched using the Mascot v2.3.2 local server against the database containing sequences of all human proteins from Refseq (71,448 proteins, release 64) and the mutated sequences constructed from WES and RNA-seq data. The target-decoy strategy was applied to maintain the FDR less than 1% at the peptide level. For the database searching using Mascot, the monoisotopic mass was used for both peptide and fragment ions, with fixed modification (Carbamidomethyl/carbamidomethylation, +57.0214 Da) on cysteine and variable modification (Oxidation, +15.9949 Da) on methionine. Tryptic cleavage after Lys or Arg was selected and up to 2 missed cleavage sites were allowed. The precursor and fragment ion mass tolerance 20 ppm and 0.05 Da.

A normalized label-free quantitation method based on the extracted ion chromatograms (XICs) was applied to all confidently identified peptides by the software of SILVER, and then the cross-search between the cancer and normal samples were performed to avoid the randomly missing in the identifications. We also used the measure of spectral count (SC) [1], which are the total number of MS/MS spectra acquired for peptides from a given protein, to quantify protein abundance.

Reference

1. Liu, H., Sadygov, R. G. & Yates, J. R. A model for random sampling and estimation of relative protein abundance in shotgun proteomics. *Anal. Chem.* **76,** 4193–4201 (2004).

Supplementary figure legends

Supplementary Figure S1. Analysis of depth of coverage. a, the distribution of depth indicated that approximate 0.25% sequences were not covered by WES; and , the cumulative depth of coverage showed that >99% bases in the captured regions were covered at least eight times (depth ≥ 8). The distribution of depth of coverage did not differ among three sequenced tissues (ANOVA, *P*=0.84).

Supplementary Figure S2. Functional classification of somatic mutations identified by WES (a) and RNA-centric variants in the combined RNA-seq data sets (b).

Supplementary Figure S3. Functional pathway analysis of 237 genes using IPA. (a) Pathway analysis indicated that 10 functions and diseases were related to liver. The number in the parenthesis is the number of genes for each disease and function. (b) In the network, genes related to liver disease and function; genes were highlighted if their tumor-mutated allele was translated.

Supplementary Figure S4. Validation of a mutated amino acid in *HNF1A* (chr12:121431992, NP_000536, p.S247T) by SRM. (a) The mutated amino acid (T); and (b) The wild-type amino acid (S).

Supplementary Figure S5. A pipeline for identification of somatic mutations

Supplementary Figure S6. A pipeline for calling and filtering sequence variants in RNA-seq

Supplementary table legends

Supplementary Table S1. Non-silent somatic mutations in DNA mismatch repair genes

Supplementary Table S2. Summary of WES reads in the studied patient

Supplementary Table S3. The total number of reads and mapped reads in two independent RNA-seq experiments (mRNA_R1 and mRNA_R2)

Supplementary Table S4. Summary of mass spectrometry in two independent proteome profiling experiments (Pro_R1 and Pro_R2)

Supplementary Table S5. Number of genes and somatic mutations expressed at the transcriptome level in two independent experiments of RNA-Seq and the merged data

Supplementary Table S6. Allelic fraction of somatic mutations in potential HCC driver genes

Supplementary Table S7. Allelic fraction of somatic mutations in subtle cancer driver genes

Supplementary Table S8. Allelic fraction of somatic mutations in the genes related to liver diseases and functions by Ingenuity Pathway Analysis (IPA)

Supplementary Table S9. Targeted peptides for selective reaction monitoring (SRM) detection

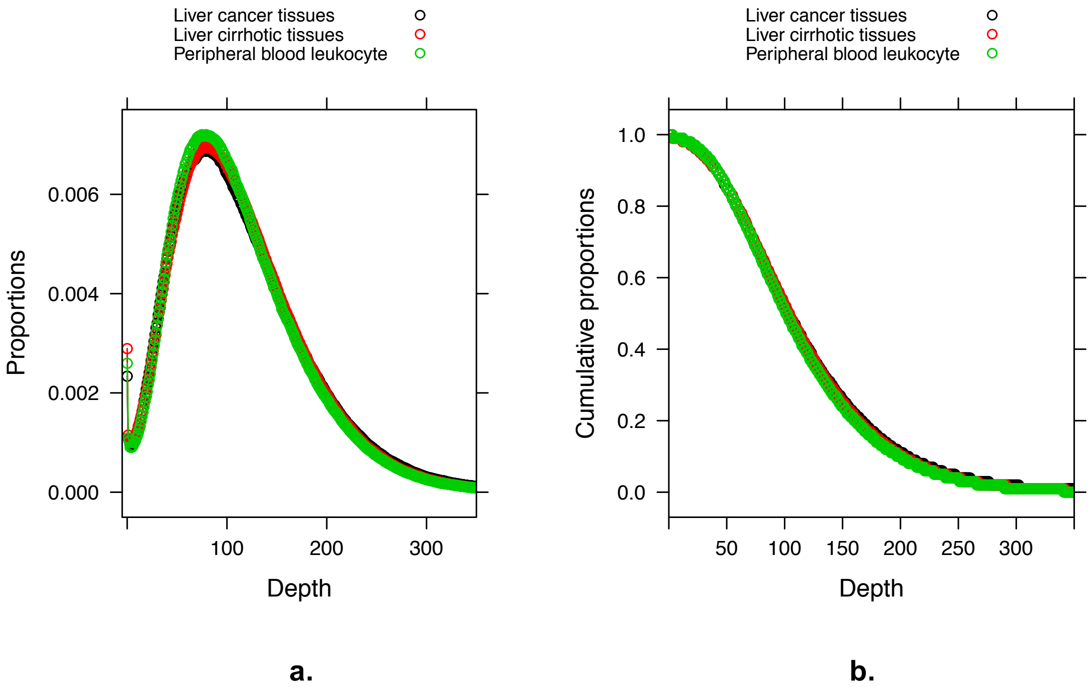

Supplementary Figure S1

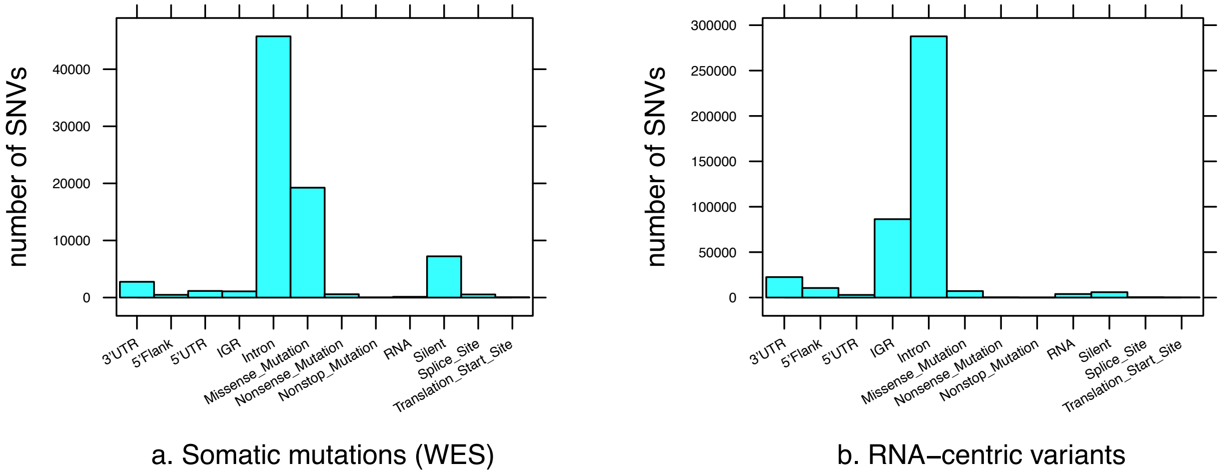

Supplementary Figure S2

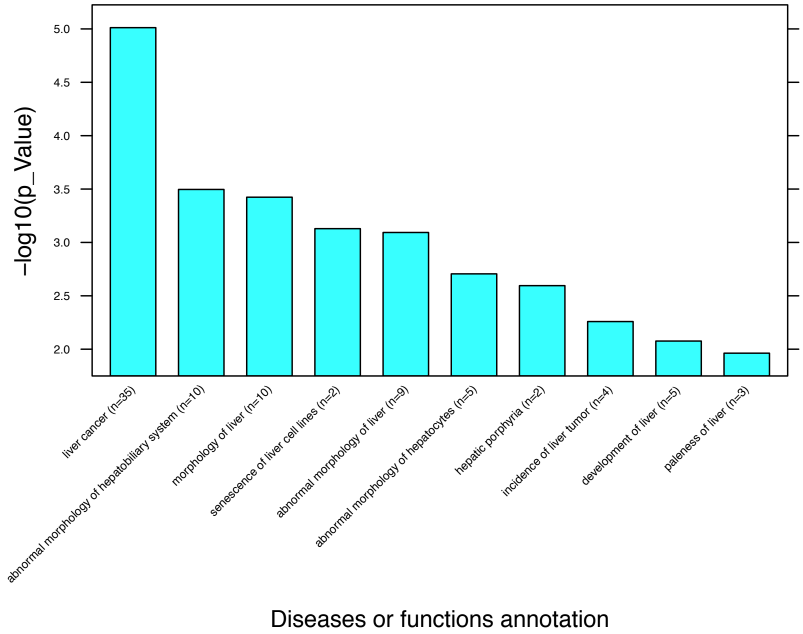

a

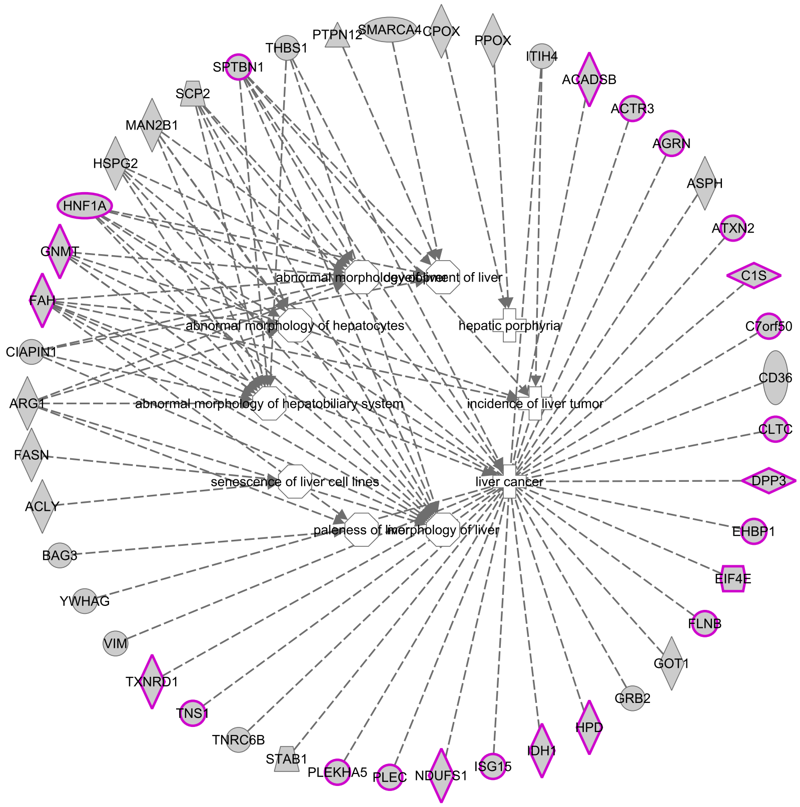

b

Supplementary Figure S3

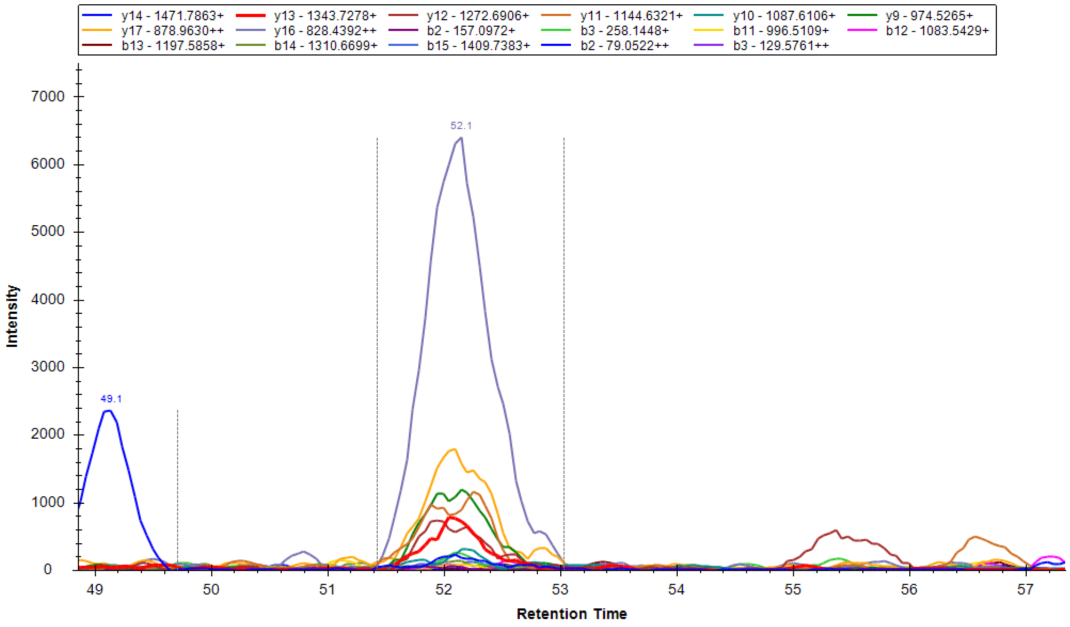

a. Mutant allele

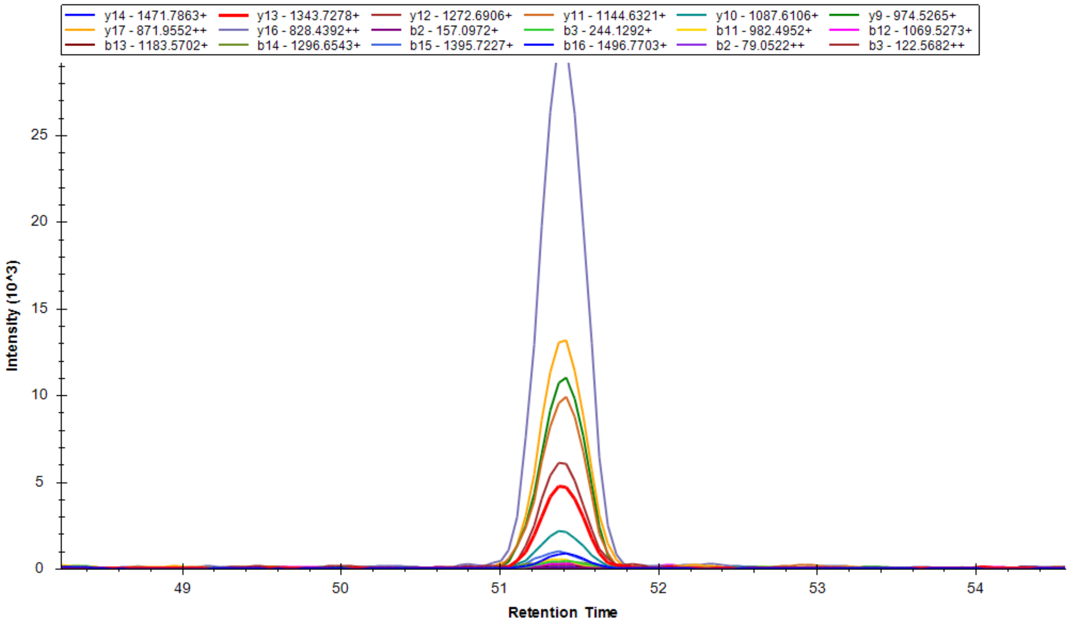

b. Wild type allele

Supplementary Figure S4

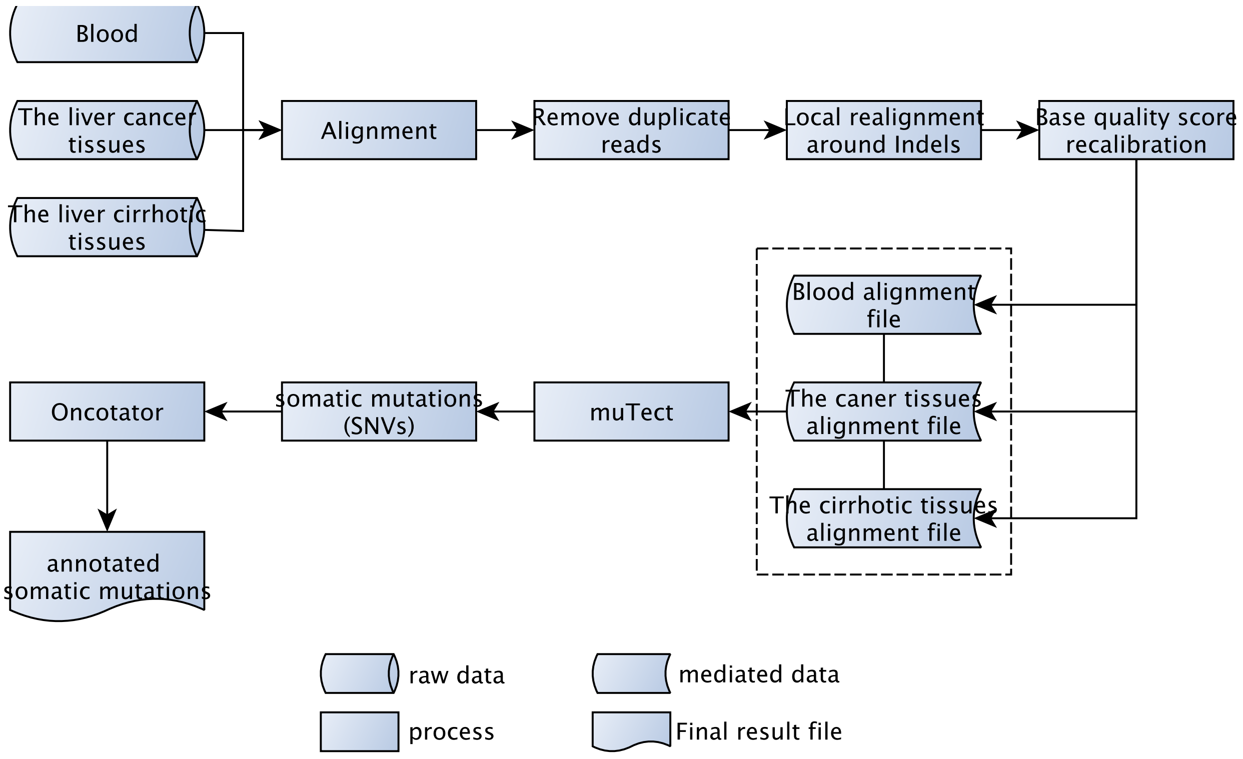

Supplementary Figure S5

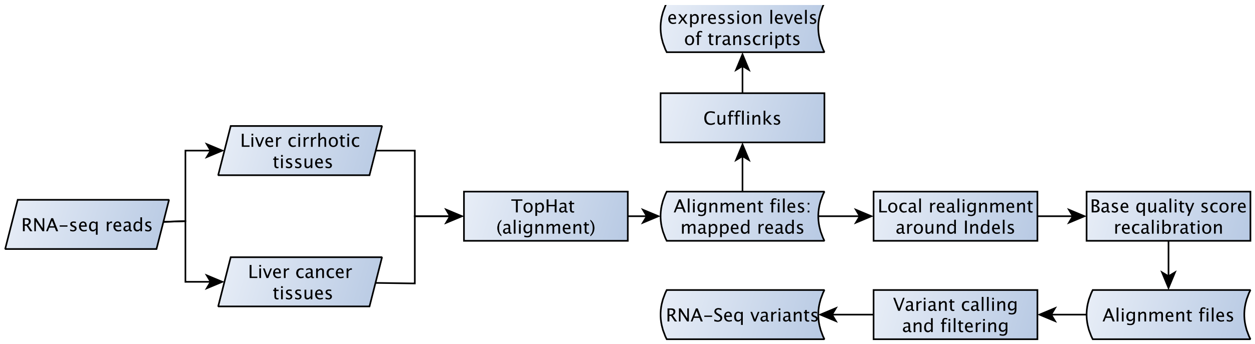

Supplementary Figure S6

Supplementary Table S1. Nonsilent somatic mutations identified in DNA mismatch repair genes in the studied patient

Chr:Pos	rs ID	Accession	AA Change	Polyphen	Symbol
2: 47630358	rs63751099	NP_000242	Q10*	Non-sense	*MSH2*
2: 47709981	—	NP_000242	S900T	Benign	*MSH2*
2: 48027584	—	NP_000170	L821P	Probably damaging	*MSH6*
2: 48027758	—	NP_000170	A879V	Benign	*MSH6*
6: 31712037	—	NP_751898	N203S	Benign	*MSH5*
7: 6026747	—	NP_000526	D550G	Benign	*PMS2*
14: 75508356	—	NP_001035197	S1143P	Probably damaging	*MLH3*
14: 75513519	—	NP_001035197	F947S	Benign	*MLH3*

MSH2, mutS homolog 2; *MSH6*, mutS homolog 6; *MSH5*, mutS homolog 5; *PMS2*, PMS2 postmeiotic segregation increased 2 (*S. cerevisiae*); *MLH3*, mutL homolog 3

Supplementary Table S2. Summary of WES reads in the studied patient

Run	Total	Mapped	Rate
Liver cirrhotic tissues			
BJ22N_L4	53,544,768	53,168,665	0.9930
BJ22N_L5	53,210,454	52,838,341	0.9930
Liver cancer tissues			
BJ22T_L5	56,115,372	55,763,527	0.9937
BJ22T_L6	55,616,690	55,269,482	0.9937
Peripheral blood leukocyte			
BJ22P_L3	52,515,626	52,264,300	0.9952
BJ22P_L4	52,456,168	52,203,965	0.9952

Supplementary Table S3. Number of reads and mapped reads in two independent RNA-Seq replicates

Replicate	Run	Total reads	Mapped reads[1]	Rate
mRNA_R1	The Liver cirrhotic tissue			
	BJ22N_L1	67,410,194	62,256,326	0.9235
	BJ22N_L6	15,819,056	14,621,992	0.9243
	BJ22N_L7	15,818,706	14,622,880	0.9244
	BJ22N_L8	15,869,748	14,673,681	0.9246
	The Liver cancer tissue			
	BJ22T_L1	65,140,898	58,148,829	0.8927
	BJ22T_L4	47,772,662	42,721,439	0.8942
mRNA_R2	The Liver cirrhotic tissue			
	BJ22R2N_L2	131,662,802	121,059,668	0.9197
	The Liver cancer tissue			
	BJ22R2T_L2	128,985,984	121,585,182	0.9426

Supplementary Table S4. Summary of mass spectrometry in two independent proteome profiling replicates

	Pro_R1	Pro_R2	Combined (Pro_R1+Pro_R2)
Mass spectra	814,387	607,754	1,422,141
Unique peptides	77,070	85,785	98,696
Non-redundant proteins (FDR<1%)	8,311	8,763	9,255
Peptide ion spanning mutation site	2,578	2,919	3,442
Number of mutations	1,191	1,348	1,517
Peptide ion spanning somatic mutation	922	1084	1,279
Number of somatic mutations	415	489	546

Supplementary Table S5. Number of genes and tumor-mutated alleles expressed at the transcriptome levels in two independent RNA-Seq replicates and the combined data

Patterns	mRNA_R1			mRNA_R2			Combined (R1 + R2)		
	Genes	Mutations	%[3]	Genes	Mutations	%[3]	Genes	Mutations	%[3]
w/o_expression[1]	426	518	10.4	490	604	12.13	348	425	8.53
w/o_covered[2]	1072	1302	26.14	850	1087	21.83	643	806	16.18
Wild-type allele	1641	1891	37.97	1333	1508	30.28	1461	1688	33.90
Both alleles	1047	1188	23.86	1460	1673	33.59	1683	1981	39.78
Mutant-type allele	81	81	1.63	107	108	2.17	79	80	1.61

[1]: Mutation sites in genes that was not expressed at the levels of transcriptome; w/o, without

[2]: Mutation sites were not covered by RNA-Seq

[3]: Percentage of somatic mutations

Supplementary Table S6. Allelic fraction of somatic mutations in potential HCC driver genes

Symbol	Chr_pos	Allelic fraction			Site depth		
		DNA	RNA	Protein	DNA	RNA	Protein
ARID1A	chr1:27056157	0.29	0	NA	193	41	NA
CDKN2A	chr9:21971035	0.24	0.31	NA	159	16	NA
ARID2	chr12:46205331	0.10	0	NA	50	8	NA
BCL9	chr1:147091031	0.42	NA	NA	149	NA	NA
NFE2L2	chr2:178095533	0.43	0.23	NA	151	190	NA
ATM	chr11:108175552	0.10	0	0	62	16	1
SMARCA4	chr19:11097111	0.43	0.41	0	97	80	3
TSC2	chr16:2125814	0.45	0.24	NA	124	115	NA
APC	chr5:112170817	0.50	0.54	NA	44	26	NA
JAK2	chr9:5044423	0.08	0	NA	48	12	NA
HNF1A	chr12:121431992	0.44	0.55	0.56	142	96	9

Shared is the prioritized personalized mutation-drivers in HCC

Supplementary Table S7. Allelic fraction of somatic mutations in subtle cancer driver genes

Symbol	Chr_pos	Allelic fraction			Site depth		
		DNA	RNA	Protein	DNA	RNA	Protein
NOTCH2	chr1:120459074	0.04	0	NA	139	245	NA
NOTCH2	chr1:120468015	0.34	0.37	NA	164	159	NA
ARID1A	chr1:27056157	0.29	0	NA	193	41	NA
JAK1	chr1:65321212	0.43	0.41	NA	56	249	NA
FUBP1	chr1:78435648	0.06	0	0	99	73	4
NFE2L2	chr2:178095533	0.43	0.23	NA	151	190	NA
IDH1	chr2:209104698	0.35	0.4	0.22	62	249	9
DNMT3A	chr2:25470020	0.40	0	NA	127	4	NA
DNMT3A	chr2:25523022	0.43	0	NA	88	4	NA
ALK	chr2:29443601	0.54	0	NA	56	2	NA
MSH2	chr2:47630358	0.45	NA	NA	31	NA	NA
MSH2	chr2:47709981	0.17	0	NA	47	3	NA
MSH6	chr2:48027584	0.40	0.42	NA	156	175	NA
MSH6	chr2:48027758	0.43	0.37	NA	190	38	NA
SETD2	chr3:47158212	0.43	0.56	NA	82	48	NA
PBRM1	chr3:52610613	0.38	0.21	NA	97	42	NA
PBRM1	chr3:52661322	0.44	0.33	NA	88	93	NA
PBRM1	chr3:52662914	0.39	0.53	NA	62	70	NA
TET2	chr4:106157905	0.39	0.54	NA	157	28	NA
TET2	chr4:106158245	0.08	0	NA	102	48	NA
FBXW7	chr4:153332474	0.35	0.37	NA	109	46	NA
APC	chr5:112170817	0.50	0.54	NA	44	26	NA
ARID1B	chr6:157511254	0.04	0	NA	226	74	NA
HIST1H3B	chr6:26032177	0.39	0.28	1	152	74	1

DAXX	chr6:33288743	0.42	0.4	NA	80	86	NA
MET	chr7:116414938	0.50	0.49	NA	12	205	NA
MLL3	chr7:151873282	0.44	0.55	NA	163	44	NA
MLL3	chr7:151878023	0.49	0.41	NA	198	39	NA
CARD11	chr7:2956953	0.40	NA	NA	104	NA	NA
NOTCH1	chr9:139393409	0.37	0	NA	278	15	NA
NOTCH1	chr9:139407503	0.08	0	NA	53	32	NA
CDKN2A	chr9:21971035	0.24	0.31	NA	159	16	NA
JAK2	chr9:5044423	0.08	0	NA	48	12	NA
FGFR2	chr10:123325159	0.47	0.25	NA	159	36	NA
ATM	chr11:108175552	0.10	0	0	62	16	1
MEN1	chr11:64572570	0.05	0	NA	105	13	NA
MEN1	chr11:64575365	0.32	0.36	NA	41	11	NA
MEN1	chr11:64575407	0.43	0.38	NA	72	16	NA
HNF1A	chr12:121431992	0.44	0.55	0.56	142	96	9
ARID2	chr12:46205331	0.10	0	NA	50	8	NA
MLL2	chr12:49420109	0.52	0.48	NA	174	48	NA
MLL2	chr12:49431982	0.45	0.22	NA	274	23	NA
MLL2	chr12:49437175	0.31	0.27	NA	83	33	NA
MLL2	chr12:49437658	0.43	0.70	NA	183	10	NA
MLL2	chr12:49445517	0.07	0	NA	109	10	NA
CREBBP	chr16:3777949	0.33	0.45	NA	55	234	NA
CREBBP	chr16:3781368	0.38	0.61	NA	72	93	0
CREBBP	chr16:3843416	0.44	0.46	NA	45	69	NA
NF1	chr17:29509573	0.32	0.20	NA	96	46	NA
BRCA1	chr17:41222980	0.38	0.33	NA	179	9	NA
BRCA1	chr17:41244639	0.04	0	NA	159	6	NA
SETBP1	chr18:42531815	0.45	0.57	NA	146	49	NA
SETBP1	chr18:42532034	0.39	0.43	NA	196	35	NA

SETBP1	chr18:42532966	0.43	0.47	NA	280	59	NA
DNMT1	chr19:10251847	0.25	0	NA	89	27	NA
SMARCA4	chr19:11097111	0.43	0.41	0	97	80	3
STK11	chr19:1220467	0.44	0.43	0	167	217	1
STK11	chr19:1221957	0.31	0.50	NA	49	169	NA
PPP2R1A	chr19:52714552	0.24	0.42	NA	46	250	NA
PPP2R1A	chr19:52716344	0.38	0.41	0	232	162	3
ASXL1	chr20:30954198	0.35	0.38	NA	134	60	NA
RUNX1	chr21:36164740	0.42	0.23	NA	65	13	NA
EP300	chr22:41545885	0.37	0.41	NA	257	106	NA
EP300	chr22:41573290	0.35	0.46	NA	300	108	NA
STAG2	chrX:123197813	0.88	0.60	0	17	134	6
BCOR	chrX:39931640	0.80	1.00	NA	41	36	NA
KDM5C	chrX:53247528	0.83	0.93	1	41	28	1

Shared are the prioritized personalized mutation-drivers in HCC

Supplementary Table S8. Allelic fraction of somatic mutations in liver-related genes by Ingenuity Pathway Analysis (IPA)

Symbol	Chr_pos	Allelic fraction			Site depth		
		DNA	RNA	Protein	DNA	RNA	Protein
PPOX	chr1:161140488	0.06	0	0	156	86	5
HSPG2	chr1:22199130	0.15	0	NA	33	212	NA
HSPG2	chr1:22214482	0.44	0	0	133	72	7
SCP2	chr1:53416522	0.38	0.57	0.10	68	223	42
ISG15	chr1:949490	0.42	0.52	0.87	81	250	15
AGRN	chr1:979308	0.49	0.36	0.12	59	33	8
GOT1	chr10:101163305	0.07	0	0	97	245	37
BAG3	chr10:121429385	0.08	0	NA	234	166	NA
BAG3	chr10:121429646	0.47	0.50	0.78	85	117	9
ACADSB	chr10:124813258	0.36	0.49	0.38	148	249	219
VIM	chr10:17271461	0.06	0	0	147	249	18
DPP3	chr11:66259041	0.49	0.41	0	139	69	23
TXNRD1	chr12:104712799	0.38	0.38	0.19	104	250	43
ATXN2	chr12:111951187	0.39	0.41	0.33	279	68	3
HNF1A	chr12:121431992	0.44	0.55	0.56	142	96	9
HPD	chr12:122294503	0.36	0.51	0.61	118	250	23
PLEKHA5	chr12:19440438	0.43	0.59	0	72	68	7
PLEKHA5	chr12:19512464	0.55	0.50	0	128	117	1
C1S	chr12:7177786	0.43	0.40	0.10	138	250	10
THBS1	chr15:39874936	0.46	0	0	57	26	8
FAH	chr15:80473495	0.50	0.51	0.57	40	247	21
CIAPIN1	chr16:57468042	0.42	0.41	0.43	105	118	7
ACLY	chr17:40042483	0.38	0.42	0	60	192	4

CLTC	chr17:57762458	0.60	0.45	0.46	20	248	26
GRB2	chr17:73317853	0.06	0	0	83	149	5
FASN	chr17:80038651	0.15	0	NA	106	246	NA
FASN	chr17:80041489	0.05	0	0	173	238	17
FASN	chr17:80043247	0.11	0	NA	66	249	NA
FASN	chr17:80045666	0.28	0.66	0	57	249	11
FASN	chr17:80048931	0.41	0.75	0.29	292	193	14
SMARCA4	chr19:11097111	0.43	0.41	0	97	80	3
MAN2B1	chr19:12758097	0.06	0	0	159	240	5
MAN2B1	chr19:12758339	0.07	0	NA	92	242	NA
ACTR3	chr2:114691944	0.46	0.43	0.33	50	250	12
NDUFS1	chr2:207003277	0.45	0.53	0.40	11	250	25
NDUFS1	chr2:207007461	0.06	0	0	96	236	10
IDH1	chr2:209104698	0.35	0.40	0.22	62	249	9
TNS1	chr2:218683189	0.32	0.31	NA	57	78	NA
TNS1	chr2:218755733	0.43	0.28	NA	75	50	NA
TNS1	chr2:218758271	0.42	0.32	0	12	63	12
SPTBN1	chr2:54856880	0.47	0.33	0	119	250	6
SPTBN1	chr2:54877029	0.48	0.43	0.33	146	250	3
SPTBN1	chr2:54882240	0.44	0.39	0.27	77	250	11
EHBP1	chr2:62974547	0.50	0.51	0.50	50	102	4
EHBP1	chr2:63170978	0.32	0.51	NA	95	86	NA
TNRC6B	chr22:40662093	0.05	0	0	113	52	3
STAB1	chr3:52535228	0.39	0	0	137	26	6
STAB1	chr3:52539153	0.19	0	NA	151	60	NA
STAB1	chr3:52556377	0.14	0	NA	181	45	NA
ITIH4	chr3:52860857	0.05	0	0	100	245	21
FLNB	chr3:58098028	0.30	0.42	0.42	64	200	12
CPOX	chr3:98304495	0.39	0.50	0	71	72	7

EIF4E	chr4:99806088	0.36	0.45	0.17	14	240	6
ARG1	chr6:131904899	0.05	0	0	186	247	118
GNMT	chr6:42928545	0.47	0.38	0.43	43	201	7
C7orf50	chr7:1049717	0.40	0.51	0.43	89	200	7
YWHAG	chr7:75959315	0.04	0	0	349	247	13
PTPN12	chr7:77210797	0.06	0	NA	48	42	NA
PTPN12	chr7:77221550	0.13	0.23	0	100	64	3
CD36	chr7:80302125	0.44	0	0	43	152	11
PLEC	chr8:144992371	0.58	0.39	0.15	33	84	40
PLEC	chr8:144993134	0.37	0.47	0	253	187	7
PLEC	chr8:144995359	0.41	0.52	0.30	135	79	10
PLEC	chr8:144998111	0.41	0.59	0	117	56	1
ASPH	chr8:62465634	0.37	0.47	0	115	237	14
ASPH	chr8:62479759	0.16	0	NA	116	37	NA

Shared are the prioritized personalized mutation-drivers in HCC

Supplementary Table S9. Targeted peptides for selective reaction monitoring (SRM) detection

Chr_pos	Symbol	Accession	Protein change	Synthesized peptides
chr11:66259041	*DPP3*	NP_569710.2	p.A292V	AYAANSHQGQMLAQYIESFTQGSIEAHK
chr12:7177786	*C1S*	NP_001725.1	p.G633D	GMDSCKGDSDGAFAVQDPNDK<br>GMDSCKGDSGGAFAVQDPNDK
chr8:144992371	*PLEC*	NP_000436.2	p.L4010P	RDDGTGQPLLPLSDAR<br>RDDGTGQLLLPLSDAR
chr1:949490	*ISG15*	NP_005092.1	p.R44C	IGVHAFQQCLAVHPSGVALQDR<br>IGVHAFQQR
chr12:104712799	*TXNRD1*	NP_003321.3	p.A280V	VVYENVYGQFIGPHR<br>VVYENAYGQFIGPHR
chr12:121431992	*HNF1A*	NP_000536.5	p.S247T	GVTPSQAQGLGSNLVTEVR<br>GVSPSQAQGLGSNLVTEVR
chr12:57350937	*RDH16*	NP_003699.3	p.K104E	DEGLWGLVNNAGISLPTAPNELLTK<br>DKGLWGLVNNAGISLPTAPNELLTK
chr15:80473495	*FAH*	NP_000128.1	p.I392V	FLLDGDEVIVTGYCQGDGYR<br>FLLDGDEVIITGYCQGDGYR
chr17:57762458	*CLTC*	NP_004850.1	p.N1492K	TSIDAYDNFDK<br>TSIDAYDNFDNISLAQR
chr2:207003277	*NDUFS1*	NP_004997.4	p.Y442N	VALIGSPVDLTYTNDHLGDSPK<br>VALIGSPVDLTYTYDHLGDSPK
chr2:54877029	*SPTBN1*	NP_003119.2	p.K1827R	RLPEELGR<br>KLPEELGR
chr2:54882240	*SPTBN1*	NP_003119.2	p.N1952K	DVSSVELLMNK<br>DVSSVELLMNNHQGIK
chr3:58098028	*FLNB*	NP_001448.2	p.Y910H	HTPTQQGNMQVLVTYGGDPIPK<br>YTPTQQGNMQVLVTYGGDPIPK
chr8:144995359	*PLEC*	NP_000436.2	p.T3014M	CVEDPEMGLCLLPLTDK<br>CVEDPETGLCLLPLTDK

Shared are the prioritized personalized mutation-drivers in HCC
